# Enhanced Thermoelectric Performance of Cu_2_SnSe_3_-Based Composites Incorporated with Nano-Fullerene

**DOI:** 10.3390/ma9080629

**Published:** 2016-07-28

**Authors:** Degang Zhao, Jiai Ning, Di Wu, Min Zuo

**Affiliations:** School of Materials Science and Engineering, University of Jinan, 336 Nan-Xinzhuang West Road, Jinan 250022, China; crystal4885@sina.com (J.N.); 18366100143@163.com (D.W); mse_zuom@ujn.edu.cn (M.Z.)

**Keywords:** thermoelectric alloy, composites, Cu_2_SnSe_3_, C_60_

## Abstract

In this study, nano-sized fullerene C_60_ powder was sufficiently mixed with Cu_2_SnSe_3_ powder by ball milling method, and the C_60_/Cu_2_SnSe_3_ composites were prepared by spark plasma sintering technology. The fullerene C_60_ distributed uniformly in the form of clusters, and the average cluster size was less than 1 μm. With increasing C_60_ content, the electrical conductivity of C_60_/Cu_2_SnSe_3_ composites decreased, while the Seebeck coefficient was enhanced. The thermal conductivity of composites decreased significantly, which resulted from the phonon scattering by the C_60_ clusters located on the grain boundaries of the Cu_2_SnSe_3_ matrix. The highest figure of merit *ZT* of 0.38 was achieved at 700 K for 0.8% C_60_/Cu_2_SnSe_3_ composite.

## 1. Introduction

Thermoelectric (TE) materials have attracted increasing worldwide attention due to their potential application in electronic cooling, waste heat recovery, and power generation [1,2,3,4]. The conversion efficiency of TE materials is determined by the dimensionless figure of merit, *ZT* = α^2^σ*T*/*κ*, where α is the Seebeck coefficient, σ is the electrical conductivity, *T* is the absolute temperature, and *κ* is the total thermal conductivity. The total thermal conductivity is composed of an electron part (*κ_E_*) and a phonon part (*κ_L_*). Therefore, to maximize the *ZT* value of TE materials, a large α and σ, as well as a low κ are required. In recent years, several classes of bulk materials with high *ZT* have been discovered and developed, such as skutterudites, clathrates, and Cu-based chalcogenide semiconductors.

Cu-based chalcogenide compounds with a diamond-like structure, such as ternary Cu_2_MSe_3_ (M = Sn, Ge) and Cu_3_SbSe_4_, have attracted a lot of attention recently, due to their quite low thermal conductivity. In several Cu-based chalcogenide compound systems, the Cu_2_SnSe_3_ structure has partial “phonon glass electron crystal” (PGEC) characteristic, which makes it possible to achieve high TE performance. The Cu-Se bond network dominates the electron conduction, while the contribution from the element Sn is very weak; thus, the Sn site is suitable for optimization of the TE property. Various attempts, including doping by partial substitution, have been made to improve the thermoelectric properties of Cu_2_SnSe_3_ compound [5,6,7]. Shi et al. have reported the In-doped Cu_2_In*_x_*Sn*_1-x_*Se_3_ and the maximum *ZT* value reaches 1.14 at 850 K for Cu_2_In_0.1_Sn_0.9_Se_3_ sample [8]. Fan et al. fabricated the Cu_2_Ga*_x_*Sn*_1-x_*Se_3_ samples using hot-press sintering technique and achieved the *ZT* value of 0.43 for Cu_2_Ga_0.075_Sn_0.925_Se_3_ sample [9]. In addition, Skoug, et al. also confirmed that doping with isoelectronic Ge on the Sn site is also effective in enhancing the *ZT* value [10]. Aside from doping, the dispersion of nanostructure phases into the thermoelectric matrix is also an attractive approach to improving the performance of TE materials. However, the Cu_2_SnSe_3_-based thermoelectric composites are scarcely investigated, because the enhancement of *ZT* is unapparent compared with the doping. Although significant reduction in the lattice conductivity can be achieved via enhanced phonon scattering at grain boundaries or matrix/inclusion interfaces, the electrical conductivity of TE composites also decreases, resulting in a marginal improvement of the overall *ZT* value. In addition, good selection of the dispersed phase and the control of microstructure are also required for TE composite [11,12,13,14]. Therefore, effective enhancement of *ZT* for TE composites depends on the microstructure of composites—i.e., the distribution or the shape of the component.

Fullerene C_60_ has very high elastic modulus and is a chemically-stable nonpolar fullerene molecule. C_60_ and C_60_-decorated grain boundaries may provide an effective phonon scattering, which could decrease the lattice thermal conductivity. Meanwhile, the scattering of charge carriers (electrons or holes) by the C_60_ could be ineffective due to the large value of electron (hole) wavelength compared to a fullerene molecule size. Blank and Kulbachinskii, et al. reported that the addition of 0.5 vol % C_60_ improved the TE properties of Bi_0.5_Sb_1.5_Te_3_ material, and the *ZT* value obtained was 1.17 at 450 K [15,16]. Shi, et al. found that adding 6.5 mass% C_60_ into pure CoSb_3_ can increase the *ZT* value, while adding amounts between 0.5% and 4.8% into CoSb_3_ decreased the *ZT* value [17]. The similar results of Itoh, et al. showed that the maximum *ZT* value for the 1% C_60_/Co_0.92_Ni_0.08_Sb_2.96_Te_0.04_ composite was 0.62 at 800 K, which was evidently higher than that of C_60_-free sample [18]. Nandihalli, et al. also reported that the *ZT* value of C_60_/Ni_0.05_Mo_3_Sb_5.4_Te_1.6_ composites was enhanced in the whole temperature range due to the large decrease of *κ_L_* [19]. In this contribution, we attempted to introduce the C_60_ into a Cu_2_SnSe_3_ system and expected to achieve a larger reduction in the thermal conductivity of C_60_/Cu_2_SnSe_3_ composites.

In the present work, the fullerene C_60_ powder was incorporated into Cu_2_SnSe_3_ matrix using ball milling (BM), and C_60_/Cu_2_SnSe_3_ composites were fabricated by spark plasma sintering (SPS) technology. Effects of C_60_ particles on the thermoelectric properties of C_60_/Cu_2_SnSe_3_ composites were discussed, and the results are beneficial to the development of Cu_2_SnSe_3_-based composites with high performance using BM-SPS technology.

## 2. Experimental Procedures

**Figure 1:**
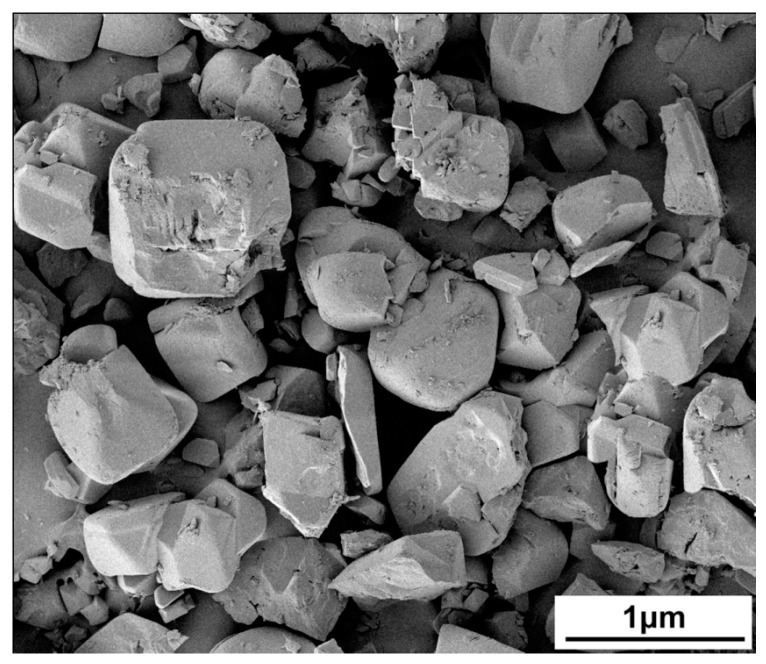
SEM image of fullerene powder with average particle size of 500 nm.

The polycrystalline Cu_2_SnSe_3_ samples were synthesized by melting method. The stoichiometric amount of starting materials Cu (powder, 99.95%), Sn (powder, 99.999%), and Se (shot, 99.999%) were first placed in a carbon crucible enclosed in evacuated fused-silica ampoules. The ampoules were slowly heated to 1173 K and held for 12 h in a vertical furnace. Then, the ampoules were slowly cooled to 873 K in 24 h, followed by annealing at this temperature for 2 days. Finally, the obtained ingots were ground into fine powder. Commercially available fullerene powder with average particle size of 500 nm (XFNANO, Nanjing, China) was chosen as the nanoinclusion, as shown in Figure 1. The fullerene C_60_ purity is 99.98%, and the other 0.02% refers to impurities of C_70_ and other carbon structures. The fullerene C_60_ powder was added into the Cu_2_SnSe_3_ powder at fractions of 0.4, 0.8, 1.2, and 1.6 vol %, respectively. Then, the C_60_-added Cu_2_SnSe_3_ powders were mechanically ground with planetary ball milling equipment at 150 rpm for 240 min. The as-milled powders were sintered by spark plasma sintering (SPS 2040) at around 860 K for about 8 min under uniaxial pressure of 50 MPa in vacuum.

The density of the sintered C_60_/Cu_2_SnSe_3_ composites was measured using the Archimedes method. The constituent phases of the samples were determined by X-ray diffractometry (Cu K*_α_*, Rigaku, Rint2000, Tokyo, Japan). The chemical composition of bulk samples was characterized using electron probe micro-analysis (EPMA, JEOL, JXA-8100, Tokyo, Japan) with a wavelength dispersive spectrometer (WDS). The composition was calculated by averaging five spots. The microstructure of all C_60_/Cu_2_SnSe_3_ composites was observed by high-resolution transmission electron microscopy (HRTEM, JEM2100F, JEOL, Tokyo, Japan). The thermal diffusivity (λ) was measured by laser flash method (Netzsch LFA427) in a flowing Ar atmosphere between 300 and 700 K. The thermal conductivity was calculated from the relationship *κ* = *ρ*λ*C_p_*, where *ρ* is the density of the sintered sample and *C_p_* is the specific heat capacity. The electrical conductivity and Seebeck coefficient were measured simultaneously using commercial equipment (ZEM-3, ULVAC-RIKO, Tokyo, Japan) on a bar-type sample with a dimension of 2 × 2 × 10 mm^3^. The Hall coefficient (*R*_H_) was measured using the van der Pauw’s method in vacuum with a magnetic field of 2 T. The carrier concentration (*p*_H_) and mobility (μ_H_) were estimated from the relations of *p*_H_ = 1/(*eR*_H_) and μ_H_ = σ*R*_H_, based on the assumption of single band model, where *e* is the electronic charge. All measurements were performed in a temperature range of 300–700 K.

## 3. Results and Discussion

### 3.1. Microstructure and XRD Analysis

**Figure 2:**
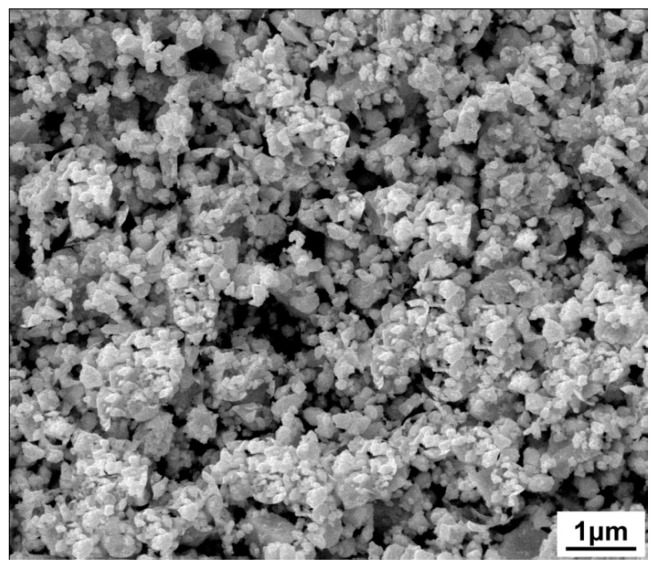
SEM image of the 1.6 vol % C_60_/Cu_2_SnSe_3_ powder after ball milling.

**Figure 3:**
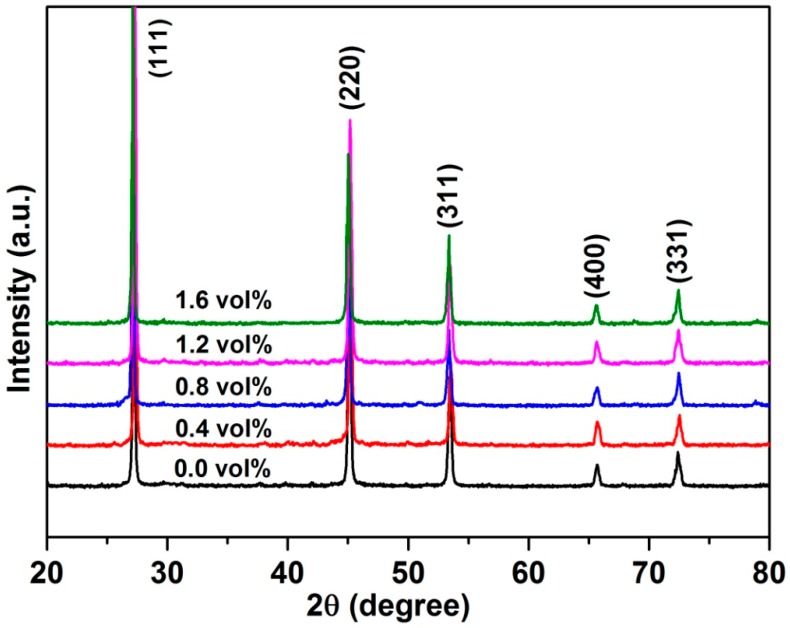
XRD patterns of sintered C_60_/Cu_2_SnSe_3_ composites.

Figure 2 shows the SEM image of the 1.6 vol % C_60_-added Cu_2_SnSe_3_ powder after ball milling. It can be observed that the average particle size of milled C_60_/Cu_2_SnSe_3_ powder was about 100 nm. Figure 3 shows the X-ray diffraction patterns of *x*C_60_/Cu_2_SnSe_3_ composites (*x* = 0, 0.4, 0.8, 1.2, and 1.6 vol %) after SPS. The measured relative densities for all C_60_/Cu_2_SnSe_3_ composites after SPS are above 97% of the theoretical value. The diffraction peaks in Figure 3 are well-indexed based on the JCPDS 65-4145 (Joint Committee on Powder Diffraction Standards) of Cu_2_SnSe_3_. As the content of C_60_ in the composites is very low, the diffraction peak of C_60_ is not found in the XRD pattern of all C_60_/Cu_2_SnSe_3_ samples. Therefore, all C_60_/Cu_2_SnSe_3_ samples show the same XRD patterns with the pure Cu_2_SnSe_3_ sample.

**Figure 4:**
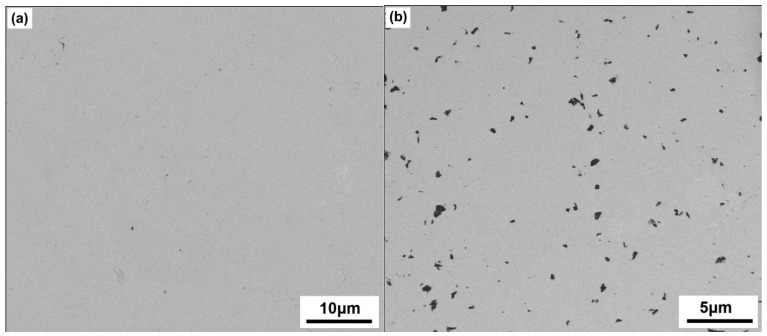
(**a**) SEM microstructure of the sintered pure Cu_2_SnSe_3_; (**b**) SEM microstructure of sintered 1.6 vol % C_60_/Cu_2_SnSe_3_ composite.

**Figure 5:**
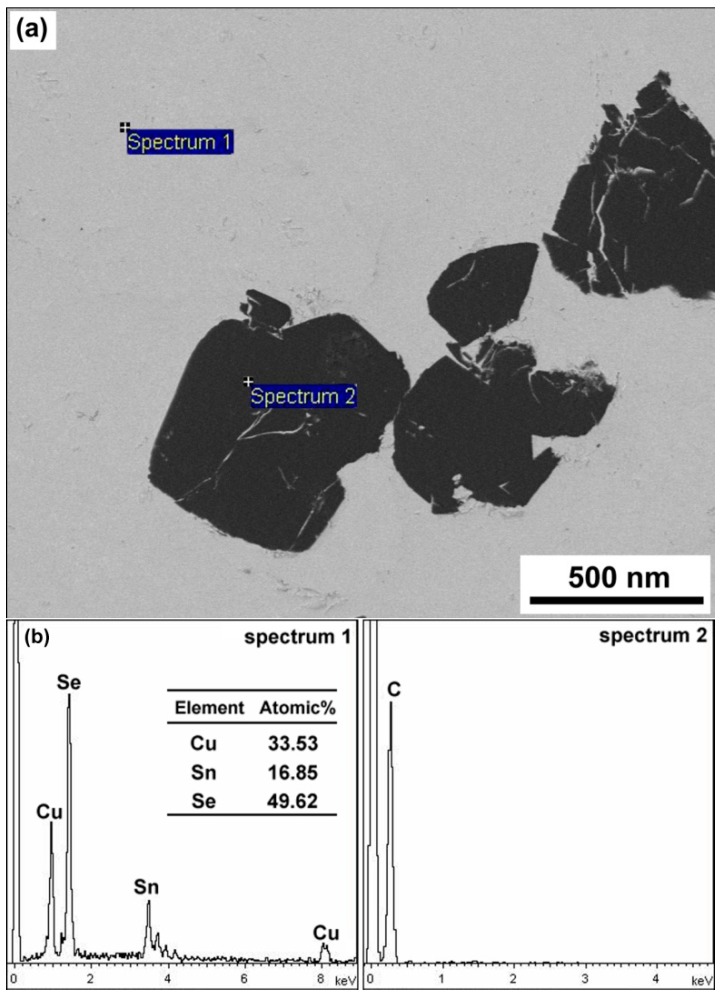
(**a**) SEM image of the sintered 1.6% C_60_/Cu_2_SnSe_3_ composite; (**b**) energy-dispersive X-ray spectroscopy (EDS) results of Cu_2_SnSe_3_ matrix and C_60_ phase.

**Figure 6:**
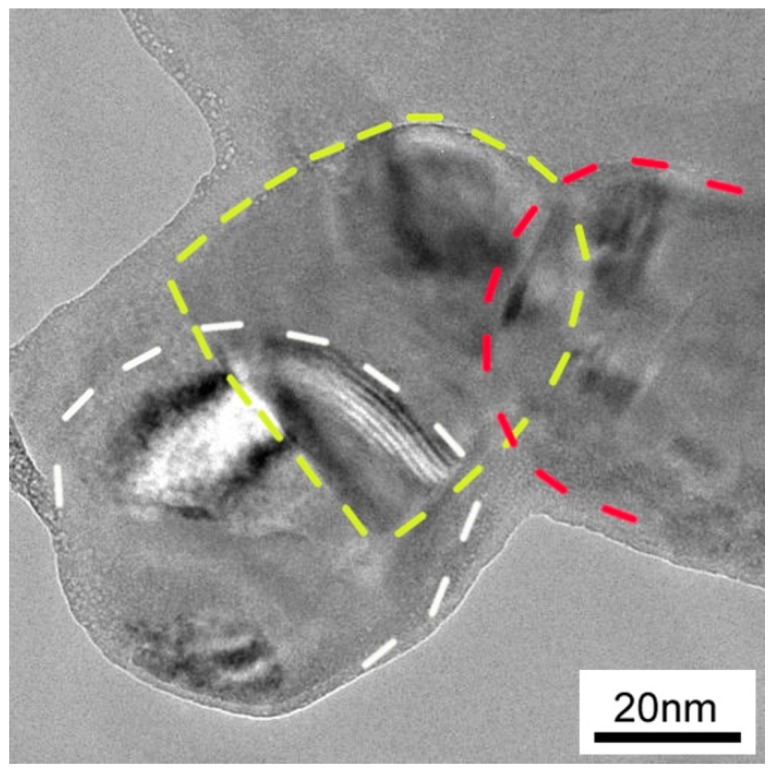
High-resolution transmission electron microscopy (HRTEM) image of a C_60_ particle in the C_60_/Cu_2_SnSe_3_ composite.

Figure 4a,b show the SEM microstructure of the sintered pure Cu_2_SnSe_3_ sample and 1.6 vol % C_60_/Cu_2_SnSe_3_ composite, respectively. The fullerene C_60_ distributed uniformly in the form of clusters, and the average cluster size was lower than 1 μm. Shi, et al. reported that in the CoSb_3_ material, most of the C_60_ molecules agglomerate into irregular micrometer-size clusters located at the grain boundaries [17]. The smaller size of clusters in this study should be due to the ball milling technology. The chemical composition of C_60_/Cu_2_SnSe_3_ composites was characterized by SEM and energy-dispersive X-ray spectroscopy (EDS), as shown in Figure 5. The results of EDS also confirm that the matrix was analyzed to be composed of 33.53 at. %; Cu, 16.85 at. %; Sn, and 49.62 at. %; Se, corresponding to the Cu_2_SnSe_3_ phase. The black phase only contains C element, indicating C_60_ phase. To further analyze the C_60_ clusters in the C_60_/Cu_2_SnSe_3_ composite, HRTEM of C_60_/Cu_2_SnSe_3_ composite was carried out, as shown in Figure 6. The size of C_60_ is about 80 nm, which means the ball milling process decreases the average size of C_60_ particles. According to the theory proposed by Faleev and Zebardaji, et al. [20,21], nano-phases that distribute in the thermoelectric matrix can result in strain fields, which could cause some changes in the band structure of the material and then greatly influence its thermoelectric properties.

### 3.2. Electrical Transport Properties

**Figure 7:**
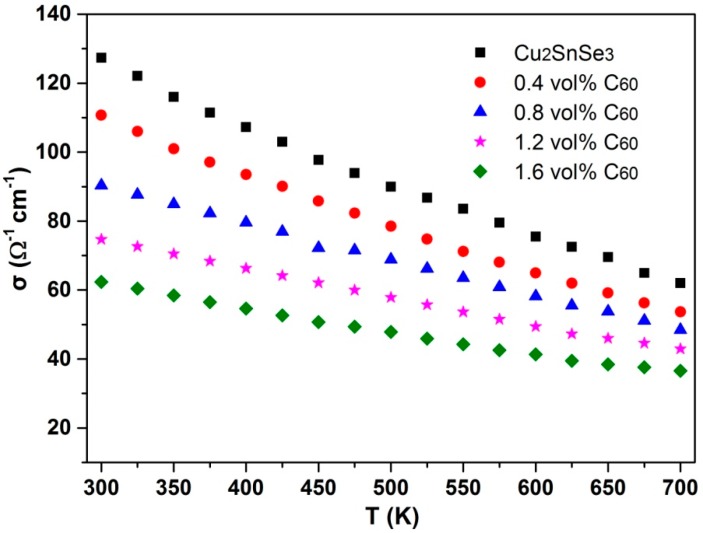
Temperature dependence of electrical conductivity (σ) of C_60_/Cu_2_SnSe_3_ composites.

Figure 7 shows the temperature dependence of electrical conductivity (σ) for C_60_/Cu_2_SnSe_3_ composites with different vol % C_60_. It can be seen that the σ of the Cu_2_SnSe_3_ matrix decreases approximately linearly with rising temperature over the measured temperature range, indicating a typical behavior of a heavily-doped semiconductor. The similar tendency of σ was also observed in C_60_/Cu_2_SnSe_3_ composites. In addition, the σ of C_60_/Cu_2_SnSe_3_ composites decreases with increasing C_60_ content, which should be attributed to the enhanced carrier scattering at the incoherent interfaces between well-dispersed C_60_ clusters and the Cu_2_SnSe_3_ matrix. Generally, in the case of carriers primarily scattered by grain barriers or interfaces between the second phase and matrix in the composites, the carrier mobility can be written as [22]

(1) $$\mu_{H}=\frac{\text{eb}}{\sqrt{8k_{B}Tm^{*}}}e^{-\frac{E_{B}}{k_{B}T}}$$

where *b* is the average grain size, *k*_B_ the Boltzmann constant, m* the carrier effective mass, and *E*_B_ the activation energy characterizing the barrier height between the matrix and the second phase. As the relative density of the *x*C_60_/Cu_2_SnSe_3_ composite is higher than 97%, the porosity effect can be eliminated. Table 1 lists some physical and structural parameters of *x*C_60_/Cu_2_SnSe_3_ composites at room temperature. As the C_60_ could act as an electron acceptor in the *p*-type C_60_/Cu_2_SnSe_3_ composite, the carrier concentration increases with increasing C_60_ content, which is consistent with the results of Blank [15]. In addition, it can be noted that the carrier mobility decreases with increasing C_60_ content. Therefore, the σ of *x*C_60_/Cu_2_SnSe_3_ composites decreases compared with the σ of the Cu_2_SnSe_3_ matrix.

**Figure 8:**
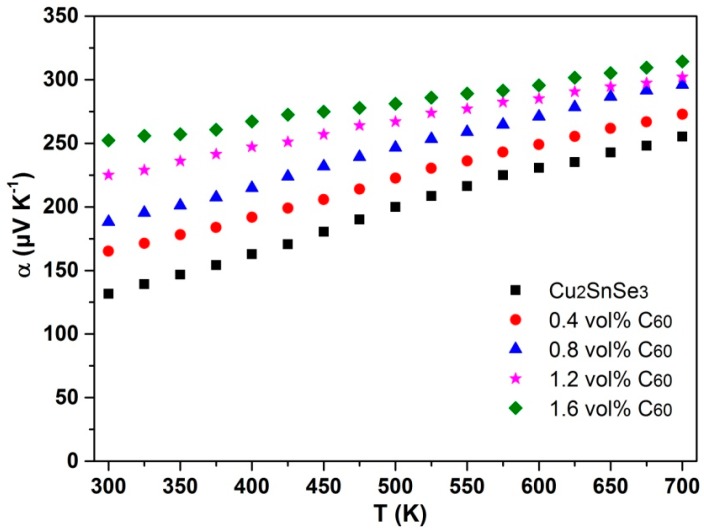
Temperature dependence of Seebeck coefficient (α) of C_60_/Cu_2_SnSe_3_ composites.

Figure 8 displays the Seebeck coefficient (α) of *x*C_60_/Cu_2_SnSe_3_ composites as a function of temperature. All composites have a positive α across the whole temperature range, indicating that the holes are major carriers. With rising temperature, the α of all *x*C_60_/Cu_2_SnSe_3_ composites increases approximately linearly and the α of 1.6% C_60_/Cu_2_SnSe_3_ composite reaches 314 μV/K at 700 K. Moreover, the α of *x*C_60_/Cu_2_SnSe_3_ composites significantly increases with the increasing content of C_60_. At room temperature, the α increases from 130 μV/K for the Cu_2_SnSe_3_ matrix to 252 μV/K for the 1.6 vol % C_60_/Cu_2_SnSe_3_ composite. The enhancement of α of *x*C_60_/Cu_2_SnSe_3_ composites should be related to the “energy filter” effect. The Seebeck coefficient can be expressed as [23],

(2) $$\alpha =-\frac{\pi }{3}\frac{k_{B}}{e}k_{B}T\frac{d\ln\sigma \left(E\right)}{dE}{|}_{E=E_{F}}-\frac{T}{n\left(E\right)}\frac{dn\left(E\right)}{dE}{|}_{E=E_{F}}$$

where *k*_B_, σ(*E*), and *n*(*E*) are Boltzmann constant, electrical conductivity, and value of density of states (DOS), respectively. Many studies have confirmed that when nano-phases or nano-inclusions are incorporated into a semiconducting matrix material, the band bending at the inclusion/matrix interface will produce a potential energy barrier which could effectively block low energy electrons, while transmitting high energy electrons [24]. This “electron energy filter” could evidently increase the local density of states near the Fermi level (*E_F_*) and enhance the Seebeck coefficient.

**Figure 9:**
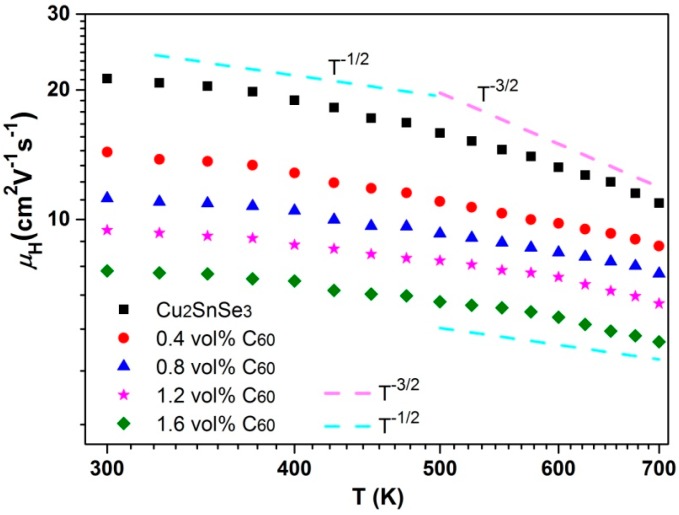
Temperature dependence of carrier mobility (μ_H_) of C_60_/Cu_2_SnSe_3_ composites.

The μ_H_ of *x*C_60_/Cu_2_SnSe_3_ composites is shown in Figure 9. The *μ*_H_ of *x*C_60_/Cu_2_SnSe_3_ composites decreases with increasing C_60_ content. In addition, the μ_H_ of *x*C_60_/Cu_2_SnSe_3_ composites was in the order of 10 cm^2^·V^−1^·s^−1^ at room temperature, which was close to that of skutterudites [25,26]. It may be caused by the similar carrier effective mass of Cu_2_SnSe_3_ and CoSb_3_ compounds. The m* can be estimated in the single parabolic band model using the following equations,

(3) $$m^{*}=\frac{h^{2}}{2k_{B}T}{\left(\frac{n}{4\pi F_{\frac{1}{2}}\left(\eta \right)}\right)}^{2/3}$$

(4) $$\alpha =-\frac{k_{B}}{e}\left[\frac{\left(r+2\right)F_{r+1}\left(\eta \right)}{\left(r+1\right)F_{r}\left(\eta \right)}-\eta \right]$$

where *F_r_*, *h*, and *r* are Fermi integral, Planck’s constant, and scattering parameter of relaxation time, respectively. The evaluated equivalent carrier effective mass of *x*C_60_/Cu_2_SnSe_3_ composites at room temperature is listed in Table 1. It can also be seen from Figure 9 that the μ_H_ of pure Cu_2_SnSe_3_ shows a temperature dependence of *T*^−1.5^ above 500 K, indicating that the acoustic phonon scattering is dominant in the temperature range from 500 to 700 K. Below 500 K, the μ_H_ of pure Cu_2_SnSe_3_ has a weak temperature dependence relationship and the relationship of μ_H_∝*T*^−0.5^ is observed, suggesting that a dominative mechanism is alloy scattering. However, the μ_H_ of *x*C_60_/Cu_2_SnSe_3_ composites deviates from the *T*^−1.5^ or *T*^−0.5^ dependence over the entire temperature range, indicating that a mixed scattering mechanism dominates these samples.

### 3.3. Thermal Transport Properties

**Figure 10:**
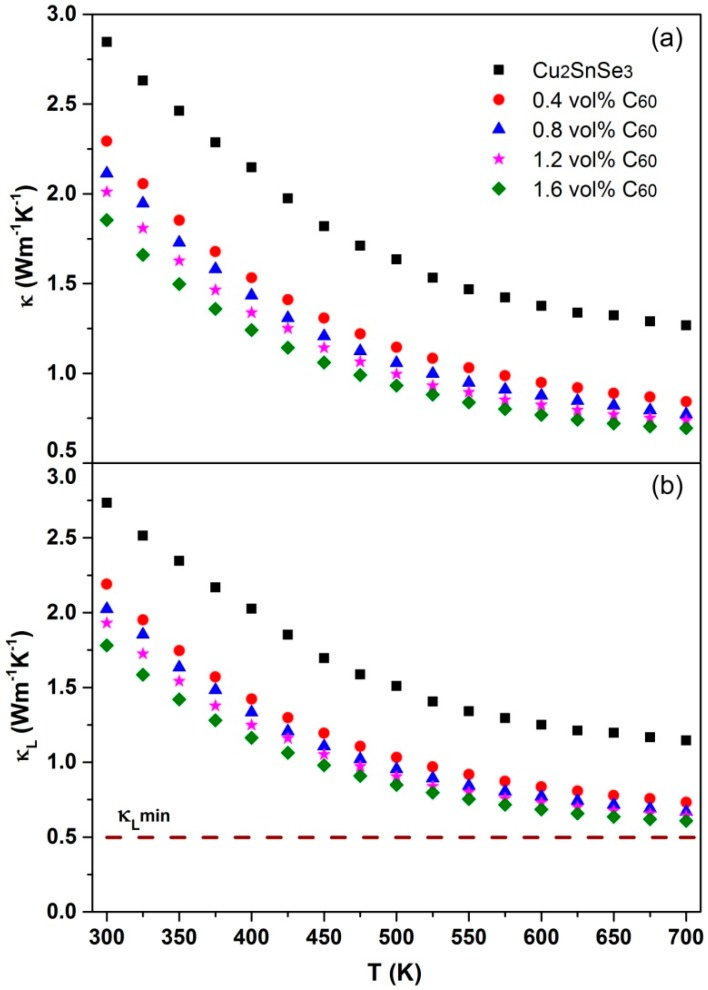
(**a**) Temperature dependence of total thermal conductivity (*κ*) of C_60_/Cu_2_SnSe_3_ composites; (**b**) temperature dependence of lattice thermal conductivity (*κ_L_*) of C_60_/Cu_2_SnSe_3_ composites.

Figure 10 displays the temperature dependence of total thermal conductivity (*κ*) and lattice thermal conductivity (*κ_L_*) for C_60_/Cu_2_SnSe_3_ composites. The *κ_L_* is estimated by subtracting the electronic contribution via the Wiedmann–Franz law (*κ_E_ = L*_0_σT, where the Lorenz number L_0_ is taken as a constant of 2.0 × 10^−8^ V^2^/K^2^) from the total thermal conductivity. The κ for all samples declines with increasing temperature. Moreover, the κ of *x*C_60_/Cu_2_SnSe_3_ composites decreases with increasing C_60_ content. The achieved *κ* of 1.6 vol % C_60_/Cu_2_SnSe_3_ composite at room temperature is 1.85 W/mK, which is 34% lower than that of pure Cu_2_SnSe_3_. The minimal *κ* of 1.6 vol % C_60_/Cu_2_SnSe_3_ composite is 0.71 W/mK at 700 K. It is well-known that the grain boundary, wide or point defects, porosity, and impurity could contribute to the decrease of *κ*. Owing to high relative density of C_60_/Cu_2_SnSe_3_ composites, the reduction of *κ* originating from the porosity is negligible. Meanwhile, the calculation of *κ_E_* shows that the reduction of *κ_E_* has a limited contribution to the decrease of *κ*. Therefore, the decrease of *κ* for C_60_/Cu_2_SnSe_3_ composites mainly originates from the depression of *κ_L_* due to the enhancement of phonon scattering by the C_60_ inclusions or nano-particles in the composite. Just as shown in Figure 10b, the *κ_L_* of C_60_/Cu_2_SnSe_3_ composites drastically decreases with the content of C_60_ increasing. The minimal *κ_L_* achieved in the present work is 0.68 W/mK at 700 K for the 1.6 vol % C_60_/Cu_2_SnSe_3_ sample, which is 43% lower than that of pure Cu_2_SnSe_3_. According to the kinetic theory [27], the minimum lattice thermal conductivity *κ_L_*_min_ can be obtained when the phonon mean free path reaches the shortest interatomic distance. The *κ_L_*_min_ can be estimated from the formula *κ_L_* = 1/3C_v_ν_m_*l*, where *C_v_* is heat capacity per unit volume of the system using Dulong and Petit value, ν_m_ the mean sound velocity, and *l* the mean free path of phonon. The ν_m_ comes from the data in reference [28]. If we assume the minimum *l* to be the interatomic distance for Cu_2_SnSe_3_ (0.238 nm), the *κ_L_*_min_ is calculated as 0.52 Wm^−1^·K^−1^, just as shown by dashed line in Figure 10b. The *κ_L_* of 1.6 vol % C_60_/Cu_2_SnSe_3_ composites approaches the *κ_L_*_min_ of Cu_2_SnSe_3_ at high temperature.

### 3.4. Figure of Merit

**Figure 11:**
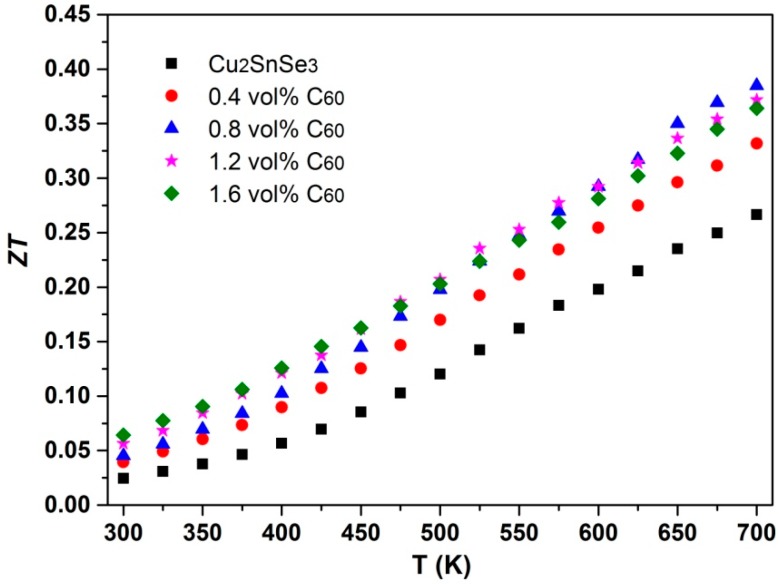
Temperature dependence of the dimensionless figure of merit of (*ZT*) of C_60_/Cu_2_SnSe_3_ composites.

The conversion efficiency of TE materials depends on the maximum dimensionless figure of merit (*ZT*). Figure 11 shows the dimensionless figure of merit (*ZT*) of C_60_/Cu_2_SnSe_3_ composites as a function of temperature. Like other doped Cu_2_SnSe_3_ investigated before [8,29,30], the *ZT* value of C_60_/Cu_2_SnSe_3_ composites increases approximately linearly with increasing temperature. Compared with the *ZT* of the Cu_2_SnSe_3_ sample, the *ZT* value of C_60_/Cu_2_SnSe_3_ composites is enhanced. For the 0.8 vol % C_60_/Cu_2_SnSe_3_sample, the maximum *ZT* value is 0.38 at 700 K, which is 45% higher than that of the pure Cu_2_SnSe_3_ sample. The enhancement of *ZT* for C_60_/Cu_2_SnSe_3_ composites is mainly attributed to the reduced *κ_L_* and the enhanced α. The addition of C_60_ into the Cu_2_SnSe_3_ matrix could improve the TE properties, which is a promising process to the design Cu-based chalcogenide compounds with high TE performance. When the material with optimized carrier concentration is selected as the matrix, the higher *ZT* value of TE composite could be achieved.

## 4. Conclusions

In this study, C_60_ was incorporated into a Cu_2_SnSe_3_ matrix, and C_60_/Cu_2_SnSe_3_ composites were fabricated using BM-SPS method. The C_60_ phase distributed uniformly in the form of clusters, and the average cluster size was less than 1 μm. With increasing C_60_ content, the electrical conductivity of C_60_/Cu_2_SnSe_3_ composites decreased, while the Seebeck coefficient of C_60_/Cu_2_SnSe_3_ composites increased due to the “electron energy filter” of the C_60_ nano-phase. The thermal conductivity of C_60_/Cu_2_SnSe_3_ composites decreased significantly, which originated from the phonon scattering by the C_60_ clusters located on the grain boundaries of the Cu_2_SnSe_3_ matrix. The maximum *ZT* of 0.38 was obtained at 700 K for 0.8 vol % C_60_/Cu_2_SnSe_3_ composite.

## Figures and Tables

**Table 1:** Chemical composition, some physical and structural parameters of *x*C_60_/Cu_2_SnSe_3_ composites at room temperature.

*x* (vol %)	Relative Density	σ (Ω^−1^·cm^−1^)	*p* (10^19^cm^−3^)	*μ*_H_ (cm^2^/Vs)	α (μV/K)	κ_L_ (Wm^−1^·K^−1^)	m* (*m_0_*)
0	98.7%	127	3.74	21.2	131	2.77	2.6
0.4	97.2%	110	4.81	14.3	165	2.22	2.8
0.8	98.9%	90	5.01	11.2	188	2.06	2.9
1.2	98.2%	75	4.98	9.4	225	1.96	3.1
1.6	97.9%	62	5.09	7.6	252	1.81	3.2
